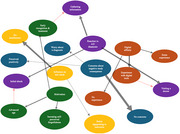# Motivation, Worries and Digital Affinity in Alzheimer's Disease Research ‐ Qualitative Findings from Davos Alzheimer's Collaborative

**DOI:** 10.1002/alz70857_100294

**Published:** 2025-12-25

**Authors:** Paulina Tegethoff, Carolin Isabella Kurz, Anna Hufnagel, Sophia Rutt, Robert Perneczky

**Affiliations:** ^1^ Department of Psychiatry and Psychotherapy, LMU Hospital, LMU Munich, Munich, Germany; ^2^ Department of Psychiatry and Psychotherapy, LMU Hospital, LMU Munich, Munich, Bavaria, Germany; ^3^ Department of Psychiatry and Psychotherapy, University Hospital, LMU Munich, Munich, Germany; ^4^ German Center for Neurodegenerative Diseases (DZNE), Munich, Germany; ^5^ Ageing Epidemiology (AGE) Research Unit, School of Public Health, Imperial College London, London, United Kingdom; ^6^ Munich Cluster for Systems Neurology (SyNergy), Munich, Germany; ^7^ Sheffield Institute for Translational Neuroscience, University of Sheffield, Sheffield, United Kingdom

## Abstract

**Background:**

Subjective cognitive deficits are the earliest clinical sign of Alzheimer's disease (AD), raising interest in early detection when disease‐modifying drugs promise the greatest benefit. The CogScreen 1 and 2 studies, part of the Davos Alzheimer's Collaborative's Healthcare Systems Preparedness program, evaluate tools for community‐based screening for AD in the earliest clinical stages. During the studies, a systematic investigation of participant motivations, concerns, and suggestions for trial improvement emerged as essential.

**Methods:**

A qualitative interview guide was developed and applied in 30 telephone interviews with randomly assigned former participants. Recorded and transcribed data were analyzed using the MAXQDA software (VERBI Software), employing an inductive‐deductive coding guide. Thematic content analysis identified key patterns and insights into the motivation, concerns and digital affinity of participants in an AD research study.

**Results:**

The study uncovered valuable perspective on Alzheimer´s research. Participants revealed diverse motivations for involvement, ranging from personal connections to dementia to aspirations for early diagnosis and treatment. Reactions to potential diagnoses ranged from shock and grief to proactive care strategies. Digital affinity varied but showed encouraging levels of familiarity with technology among older adults. Participants raised concerns about the study design, including item clarity and response scales, as well as suggestions for improving digital and traditional tools.

**Conclusion:**

By systematically capturing participant feedback, the CogScreen study provides critical insights for improving AD research methodologies, particularly in relation to the participatory nature of research. These findings underline the importance of addressing participants' motivations and fears while integrating innovative tools. The results also highlight the feasibility of digital interventions in ageing populations and emphasize trust in public institutions. Future studies should use these findings to refine early detection approaches and encourage greater participant engagement.